# Nuclear Transport of Yeast Proteasomes

**DOI:** 10.3390/biom4040940

**Published:** 2014-10-20

**Authors:** Cordula Enenkel

**Affiliations:** Department of Biochemistry, University of Toronto, Medical Sciences Building, 1 King’s College Circle, Toronto, ON M5S 1A8, Canada; E-Mail: cordula.enenkel@utoronto.ca; Tel.: +1-416-978-3843

**Keywords:** proteasome, nuclear transport, protein degradation

## Abstract

Proteasomes are conserved protease complexes enriched in the nuclei of dividing yeast cells, a major site for protein degradation. If yeast cells do not proliferate and transit to quiescence, metabolic changes result in the dissociation of proteasomes into proteolytic core and regulatory complexes and their sequestration into motile cytosolic proteasome storage granuli. These granuli rapidly clear with the resumption of growth, releasing the stored proteasomes, which relocalize back to the nucleus to promote cell cycle progression. Here, I report on three models of how proteasomes are transported from the cytoplasm into the nucleus of yeast cells. The first model applies for dividing yeast and is based on the canonical pathway using classical nuclear localization sequences of proteasomal subcomplexes and the classical import receptor importin/karyopherin αβ. The second model applies for quiescent yeast cells, which resume growth and use Blm10, a HEAT-like repeat protein structurally related to karyopherin β, for nuclear import of proteasome core particles. In the third model, the fully-assembled proteasome is imported into the nucleus. Our still marginal knowledge about proteasome dynamics will inspire the discussion on how protein degradation by proteasomes may be regulated in different cellular compartments of dividing and quiescent eukaryotic cells.

## 1. Where Are Proteins Degraded by Proteasomes?

The ubiquitin-proteasome system accounts for 80%–90% of the protein breakdown in growing yeast and mammalian cells. Its substrate repertoire comprises a large variety of short-lived proteins that have been conjugated to polyubiquitin chains [1]. Proteins associated with nuclear functions, such as cyclins and transcription factors (e.g., tumor suppressor protein p53), were among the first proteasomal substrates to be identified [2,3]. Misfolded ribosomal products were identified among the cytoplasmic proteasomal substrates, but their fraction within the substrate repertoire is significantly smaller than originally estimated [4]. Indeed, a growing body of literature demonstrates that misfolded proteins synthesized in the cytoplasm are trafficked to the nucleus for degradation (for a review, see [5]), although nuclear proteins can also be exported into the cytoplasm for degradation [6]. In this context, the finding that cytosolic proteasomes are not indispensable for protein degradation, while nuclear proteasomes are essential in yeast, challenged the concept that proteasomal proteolysis primarily occurs in the cytoplasm [7]. Although it is unknown how cytosolic misfolded proteins are targeted into the nucleus, intriguing results suggest that the canonical nuclear import pathway couples ribosome-bound nascent polypeptides to proteasomes for degradation in yeast [8]. Thus, it is not surprising that most proteasomes are indeed enriched in the nuclei of eukaryotic cells, where they exist as holo-enzymes and achieve the degradation of polyubiquitylated proteins [9,10]. Already in the early nineteen nineties, Werner Franke and colleagues located proteasomes primarily in the nuclei of *Xenopus laevis* oocytes and cultured mammalian cells [3,11,12]. Later studies on mammalian cell cultures with high cell density have drawn the attention to proteasomes in the cytoplasm [13]. To elucidate the major sites of proteasomal protein degradation, it is important understand the dynamics of proteasomes between the nucleus and the cytoplasm.

## 2. What Do We Know about Proteasome Assembly to Understand Proteasome Dynamics?

Proteasomes contain more than 33 different subunits and are composed of two major complexes, the proteolytic core (CP), with a molecular mass of ~750 kDa, and the regulator complex (RP), with a molecular mass of ~950 kDa [14]. The combination of native polyacrylamide gel electrophoresis (PAGE) and green fluorescent protein (GFP) imaging technologies results in high resolution separation of different RP-CP assemblies [15]. In dividing cells, the majority of proteasomes occur as RP-CP-RP and RP-CP holo-enzymes corresponding to 30S and 26S proteasomes, respectively. As the proteasome is second to the ribosome in terms of protein complex abundance in dividing cells, CP and RP must be continuously assembled from precursor complexes, which, despite their short-half-lives, represent a considerable fraction of newly synthesized proteins in the cell [16].

In a simplified model of CP assembly, CP precursor complexes consist of a seven-membered α subunit ring and a seven-membered β subunit ring. Certain β subunits have propeptides, the processing of which can be monitored by epitope-tagged versions of these subunits; the epitope-tag may influence the kinetics of propeptide processing [16,17], but antibodies directed against specific β-propeptides are presently unavailable.

To form the barrel-shaped CP, two CP precursor complexes, symbolized as half-CP, dimerize into the pre-holo-CP, an unstable intermediate in CP maturation. In the cavity between both inner β rings, the proteolytically active sites are exposed by auto-catalytic cleavage of the β-propeptides [18]. The correctness of CP maturation is guided by a hierarchy of several CP-dedicated chaperones, of which Ump1 has a pivotal function in the assembly of the pre-holo-CP [19]. Ump1 is degraded with the active site generation in the pre-holo-CP [17].

Access into the matured CP is inhibited by closed outer α rings [20]. Induced opening of the α ring gates is possible by intrinsically disordered proteins [21] and by the RP. In principle, the association of the RP with the CP extends the proteasomal substrate repertoire to folded proteins, which are targeted for degradation by polyubiquitylation.

The RP is composed of two subcomplexes, the RP lid and base [22]. The RP lid contains ~8 Rpn subunits, of which, Rpn11 removes the ubiquitin moieties from substrates prior to their degradation. The RP base contains 2 HEAT (Huntingtin, elongation factor 3 (EF3), protein phosphatase 2A (PP2A), yeast PI3-kinase TOR1)-like repeat proteins, called Rpn1 and Rpn2, and a ring of ATPases, which open the CP α rings and unfold the protein substrate for translocation into the CP proteolytic cavity [23]. Rpn10 and Rpn13, which confer the recognition of polyubiquitin chains, are also associated with the RP base [24,25].

## 3. Do Parallels Exist between Nuclear Transport of Proteasomes and Other Macromolecular Machineries?

The high concentration of RP-CP assemblies in the nucleus is achieved by a targeted nuclear import mechanism. The kinetics of nuclear transport of proteasomes are unknown, though they may be similar to the kinetics of the nuclear transport of ribosomes, protein complexes with comparable redundancy [26]. Two hundred thousands ribosomes are assembled during one generation of 100 min in yeast. With approximately 150 nuclear pores per cell, 1000 newly synthesized ribosomal proteins are imported per minute into the yeast nucleus, where they are assembled with ribosomal RNA into pre-ribosomal particles. Twenty five pre-ribosomal particles are exported per minute from the nucleus to the cytoplasm [26]. To achieve this efficiency, ribosome assembly and transport is orchestrated by multiple chaperones and transport receptors that belong or at least are related to the family of HEAT-repeat like β importins/karyopherins [27].

Proteasome assembly is also governed by multiple chaperones and possibly multiple transport receptors. This multiplicity might be needed for escorting proteasomal cargoes through the nuclear pore.

## 4. What Do We Know about the Basic Concept of Nuclear Transport through Nuclear Pores?

The nuclear pore is capable of maintaining an entropic permeability barrier between the nucleus and cytoplasm and prevents the random diffusion of protein cargo larger than ~40 kDa [28]. The plasticity and flexibility of this barrier is generated by intrinsically disordered FG-rich nucleoporins [29,30]. Their transient displacements are mediated by nuclear transport receptors in association with their protein cargo. The directionality of the movement of the cargo-receptor complexes through the meshwork of FG-rich nucleoporins is promoted by the Ran-GTPase gradient between the nucleus and the cytoplasm. According to the well-established model of nuclear import, the cargo:import receptor (importin/karyopherin) complex is assembled in the cytoplasm, where Ran is bound to GDP due to the Ran-GTPase activating enzyme, RanGAP. The Ran-GTP gradient confers directionality for the movement of the cargo:importin complex through the nuclear pore. In the nucleus, the cargo is released from the importin by Ran-GTP, and the Ran-GTP-bound importin is recycled back into the cytoplasm. In the case of nuclear export, the ternary cargo:exportin:Ran-GTP complex is assembled in the nucleoplasm and disassembled in the cytoplasm upon Ran-GTP hydrolysis [31]. The canonical import pathway using classical nuclear localization sequences (NLS) and the canonical NLS receptor heterodimer, importin/karyopherin αβ, named Srp1/Kap95, is well established. Protein cargos with NLS are recognized by importin/karyopherin α, while importin/karyopherin β mediates the interaction with the nuclear pore and Ran-GTP [31,32].

## 5. How Are Yeast Proteasomes Imported into the Nucleus during Cell Division?

**Figure 1:**
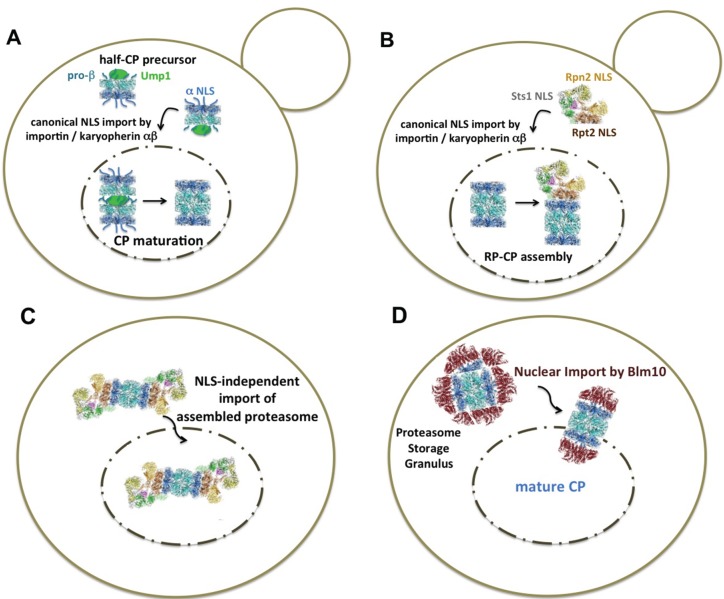
Current models of the nuclear import of yeast proteasomes (**A**) In highly proliferating cells, nuclear proteasomes are assembled from proteasomal modules that are recognized by the canonical import receptor, importin/karyopherin αβ. Classical nuclear localization sequences (NLS) are accessible in α subunits of CP precursor complexes with disordered α rings [39,40,41]. The dimerization of two half-CP yields the pre-holo-CP, a labile CP precursor complex. In the pre-holo-CP, the active sites are freed by β-propeptide processing, which is guided by the CP-dedicated maturation factor Ump1. Ump1 is degraded upon CP maturation. The α rings are closed, and the NLS is no longer accessible [17,35]. (**B**) The RP is imported into the nucleus by the canonical NLS receptor pathway. Rpt2 and Rpn2 confer NLS to the RP base and Sts1 to the RP lid. Sts1 is a short-lived protein and degraded by RP-CP assemblies [34,36,38]. (**C**) Proteasomes are imported into the nucleus as holo-enzymes independent of the canonical importin/karyopherin αβ pathway. The regulation of this recently discovered import pathway is not yet understood [47]. (**D**) When quiescent cells resume growth, nuclear import of mature CP is facilitated by Blm10, a conserved 240-kDa HEAT-like repeat protein with structural similarity to karyopherin β [48]. Blm10 preferentially binds to CP with disordered α rings, which is comparable with α ring conformations in the pre-holo-CP and half-CP. Open or disordered α rings represent an import-competent conformation [49]. CP precursor complexes are not available during quiescence, due to stalled protein synthesis. Proteasome structures were drawn according to cryo-electron microscopy studies with a license from Cell Press Elsevier [50].

In this review, I will give a historical retrospective on our knowledge about the nuclear transport of proteasomes, mainly on their nuclear import, as the nuclear export of proteasomes remains poorly understood. The dimension of the nuclear pore with an inner diameter of 39 nm [33] theoretically permits the longitudinal transport of a fully-assembled proteasome with dimensions of 20 nm × 45 nm. However, studies with yeast mutants defective in nuclear import (*srp1-49* E145K) and classical approaches in biochemistry from our and other laboratories provided evidence that nuclear proteasomes are assembled from modules in the nucleus [34,35,36,37]. Our early studies in dividing yeast cells suggested that CP precursor complexes are imported into the nucleus by the canonical importin/karyopherin αβ pathway and depend on canonical NLSs within the α subunits [35] (Table 1, Figure 1A). NLSs are not only present in several CP α subunits, but also within RP base subunits Rpt2 and Rpn2, and Sts1, a NLS-containing protein that associates with RP lid subunit Rpn11, such that each module of the proteasome is equipped with at least one NLS [38] (Figure 1B). The fusion of these NLS to non-nuclear proteins resulted in proteins that were targeted into the nucleus of digitonin-permeabilized mammalian cells. Notably, deletion of the NLS of Sts1 prevented the interaction with the canonical import receptor, importin/karyopherin α (Srp1). In this *sts1∆NLS* mutant, not only the nuclear localization of the RP lid, but also nuclear localization of the RP base and the CP was affected, suggesting that Sts1 has a general impact on nuclear localization of proteasome holo-enzymes [38]. Except for the NLS of Sts1 and the bipartite NLS of Rpn2, it was difficult to verify the functions of proteasomal NLS *in vivo* due to their redundancy, like NLSs of CP α subunits [34,35,39,40,41].

Nob1, a nuclear protein, is also involved in nuclear CP maturation and RP-CP assembly, suggesting that proteasomes reach maturation on their way to the nucleus [42]. Nuclear import of proteasomes and the nuclear proteasome mobility in dividing yeast also depend on the presence Arc3, a subunit of the actin regulatory complex, and Cdc48, an AAA-ATPase chaperone complex involved in the proteasomal degradation of unfolded proteins [43].

The model of nuclear proteasome assembly from imported modules is in agreement with Keiji Tanaka’s hypothesis, which proposed that the CP exists in two α ring conformations, with exposed NLSs or masked NLSs [44]. Most attempts to reconstitute the nuclear import of mature CP that was either isolated from *Thermoplasma acidophilum*, cultured human or yeast cells were ambiguous with regard to their interpretation [35,45,46]. In the mature CP, the NLSs within the α rings are most likely masked due to closed α ring gates [20]. The NLSs are only exposed in open α rings as apparently present within CP precursor complexes. Consistent with this concept of two CP conformations, we found that CP precursor complexes, but not mature CP, are recognized by karyopherin αβ [35].

**Table 1:** CP configurations related to nuclear transport.

Structure	Name and Specification
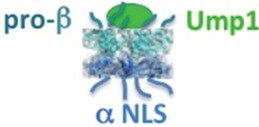	Ump1-associated CP precursor complex, symbolized as half-CP. Five of seven β subunits (cyan) are synthesized with propeptides [16,17]. Four of seven α subunits (blue) carry classical NLS (α1, α2, α4 and α6 in yeast) [39,40,41]. The α ring is disordered and can bind to Blm10 and the canonical NLS receptor importin/karyopherin αβ [35,49].
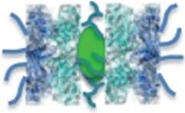	In the pre-holo-CP or nascent CP, the β subunit propeptides are processed and Ump1 is degraded [17]. The α ring is disordered and can bind to Blm10 and possibly to the NLS receptor, importin/karyopherin αβ [49].
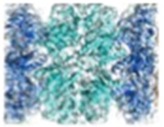	The interior between the β rings (cyan) of the mature CP harbors the proteolytic sites [18]. The α rings (blue) are closed and do not bind to Blm10 or the NLS receptor, importin/karyopherin αβ [20,35,51].
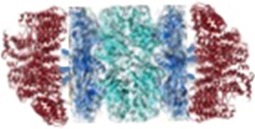	Mature CP bound to Blm10 (red) represents the nuclear import receptor-cargo complex upon exit from quiescence, when CP precursor complexes are unavailable [48]. The α rings (blue) bound to Blm10 are disordered [52].

## 6. Do Multiple Import Pathways Exist for Dividing Yeast Cells? The Canonical Import Pathway of Proteasomal Modules *vs.* the Non-Canonical Import Pathway of Holo-Enzyme Complexes

The question of proteasome import into the nucleus was recently addressed again by quantitative live-cell imaging using fluorescence correlation spectroscopy with yeast strains expressing different GFP-tagged versions of proteasomal subunits. Irrespective of which of the chosen proteasomal subunits (CP, Pre6, α4; RP base, Rpn1; RP lid, Rpn7) were labelled with GFP, each GFP-labelled subunit was fully incorporated into the respective proteasomal subcomplex and behaved as a suitable reporter protein on proteasome localization. Thus, fluorescence correlation spectroscopy enabled the monitoring of spatio-temporal dynamics of RP-CP assemblies in living yeast cells, and this promised to circumvent potential artefacts of biochemical approaches, due to labile proteasome intermediates, such as CP precursor complexes. It was found that RP-CP assemblies are stable in the importin/karyopherin α mutant, *srp1-49*, which led to the conclusion that RP-CP assembly is independent of the canonical nuclear import pathway and takes place in the cytoplasm (Figure 1C). However, one caveat of fluorescence correlation spectroscopy is that CP precursor complexes were neither detectable in wild-type nor in *srp1-49* mutants [47]. Biochemical means, such as western blot analysis for CP precursor complexes, would have given an answer as to which fraction of the CP has already matured and which fraction was still in the progress of being matured, since the use of the GFP-labeled CP subunit α4 (Pre6) may interfere with CP maturation, as indicated by delayed β propeptide processing.

How will these recent findings by fluorescence correlation microscopy be interpreted, if the short-lived and labile pre-holo-CP is the real cargo of nuclear import? Like the half-CP precursor, the pre-holo-CP also represents a CP conformation with accessible NLS. Its import could either be directly and/or indirectly mediated by the canonical NLS receptor, importin/karyopherin αβ. Accordingly, our previous attempts to isolate proteasomal import cargos from *srp1-49* mutants most likely yielded half-CP, which resulted from the decay of pre-holo-CP. The detection of incompletely processed β5 subunits (i-β5) within proteasomal import cargo points towards the presence of pre-holo-CP [35]. Redistributions of pre-holo-CP cannot be resolved by fluorescence correlation spectroscopy, because pre-holo-CP is not distinguishable from mature CP by size or shape.

Additional findings argue for the model of the nuclear import of CP precursor complexes. Ump1, the CP-dedicated maturase and reporter of CP precursor complexes, is detectable in wild-type cells, if it is stabilized by a fusion to GFP. The Ump1-GFP fusion protein is fully incorporated into CP precursor complexes and thus suited for reporting on the localization of CP precursor complexes. Ump1-GFP, like all other proteasomal subunits, primarily localized to the nucleus in dividing cells [35,49]. In the case of the *UMP1* deletion, approximately half of the CP population is composed of CP precursor complexes, most likely pre-holo-CP [53]. The inefficiency of CP maturation in the absence of Ump1 is compensated by augmented CP synthesis [54]. If one assumes that the CP is matured in the cytoplasm, GFP-labeled CP subunit β5 would then be expected to accumulate as part of CP precursor complexes in the cytoplasm of *ump1∆* cells. Instead, the opposite is observed; the nuclear CP population, which consists half of CP precursor complexes and half of mature CP, was found to be strikingly increased in *ump1∆* cells, supporting the conclusion that the majority of pre-holo-CP accumulates in the nucleus [49]. A similar phenomenon is reported for *pac1∆pac2∆* cells, in which the CP-dedicated chaperones, Pac1 and Pac2, are deleted [19]. Thus, we have reasons to believe that pre-holo-CP represents the real import cargo, despite their being impossible to track, either by classical biochemical fractionation or by advanced methods in fluorescence spectroscopy. A “molecular stethoscope” digging into the cavity of the CP would provide insight as to where the CP is matured on the road to the nucleus.

Future studies are needed to clarify the conditions under which the import of assembled RP-CP predominates the previously proposed canonical import pathway of precursor intermediates and proteasomal subcomplexes, since not only Ump1, but also RP-dedicated chaperones are present in the nucleus. It is also unclear why the cellular distribution of proteasomes was unaffected in studies using fluorescence correlation spectroscopy of the importin/karyopherin α mutant, *srp1-49*. The dependence of the nuclear import of proteasomes on the canonical pathway was called into question, even though each module of the proteasomes contains functional NLSs. The literature provides increasing evidence that the directionality and orientation of proteasome transport is governed by Sts1, a NLS-containing protein, which genetically and physically interacts with the RP lid subunit, Rpn11 [38,55]. Cut8, the fission yeast ortholog/homolog of Sts1, serves as an anchor for proteasomes at the inner nuclear membrane, but a related protein has not yet been identified in mammals [56,57].

## 7. Is the Concept of Nuclear Import of Yeast Proteasomes Applicable to Higher Eukaryotic Cells?

The question arose early whether these findings in yeast can be extended to mammalian proteasomes, since in contrast to yeast having a closed mitosis, mammalian cells undergo an open mitosis with the disintegration of the nuclear envelope. In mammalian cells, the restoration of the nuclear envelope certainly allows for nuclear reuptake of proteasomes without the need for import through nuclear pores. After cytokinesis, proteasomes were found to be primarily nuclear in daughter cells. During interphase, proteasomes were seen in clusters in the cytoplasm and at the nuclear matrix [58]. These early findings based on indirect immunofluorescence microscopy, were confirmed by later direct fluorescence microscopy using GFP-labeling techniques. The first example of a functional GFP-fusion protein of a mammalian proteasomal subunit was the cytokine-inducible iβ1 (Lmp2), which allowed the monitoring of the intracellular movements of the immune-specific CP. GFP-labeled iβ1 was found to be equally distributed throughout the nucleus and the cytoplasm of human fibrosarcoma cells [59]. Fluorescence recovery after photobleaching experiments revealed that the transport of GFP-labeled immuno-CP across the nuclear envelope, in other words through nuclear pores, occurred from the cyto- to the nucleoplasm, albeit very slowly. In fact, the presence of the immuno-CP in the nucleus seemed to depend on the mitotic breakdown of the nuclear envelope, suggesting that the immune-CP is taken up into the nucleus with the restoration of the nuclear envelope [59].

These observations are contrasted by recent findings of nuclear CP precursor complexes of cytokine-inducible immuno-CP in mouse cortical astrocytes [60]. In addition, Pomp, the mammalian ortholog of Ump1, is nuclear in human embryonic kidney cells (HEK293) [61]. In highly proliferating mammalian cells, the constitutive/standard CP is nuclear during cell proliferation. Live cell imaging studies on human melanoma cultures clearly show a primarily nuclear localization of the GFP-labeled version of the α3 subunit [62]. Thus, nuclear CP localization and maturation is not a yeast-specific phenomenon. If nuclear CP maturation is attenuated and short-lived, CP precursor complexes become detectable; nuclear CP maturation may also become traceable in mammalian cells. However, the extent of nuclear CP maturation in mammalian cells seems to depend on the cell line and the stage of the cell cycle [10].

Taken together, our knowledge about nuclear CP maturation in yeast is not simply applicable to mammalian cells, because the transition from a single-cell organism, such as yeast, to multicellular organisms and differentiated tissues is not trivial.

In contrast to yeasts, in mammalian cells, the CP precursor complex assembly was proposed to exclusively take place at the ER [63], which may provide scaffolding structures for early events of CP precursor assembly [64]. The debate about the localization of proteasome assembly and maturation is still ongoing.

## 8. How Are Yeast Proteasomes Imported into the Nucleus upon the Exit from Quiescence?

Since protein synthesis is remarkably reduced in quiescence, CP precursor complexes are not available as import cargoes. Only mature CP exists, but its nuclear import by the canonical pathway is negligible. Thus, an alternative import pathway must exist that allows nuclear import of mature CP. To resume cell growth upon the exit from quiescence, cytosolically-stored CP and RP need to be rapidly imported into the nucleus, where proteasomal proteolysis and cell cycle progression are immediately reactivated. We found that instead of the canonical import pathway, the newest member of conserved proteasome activators, Blm10 (Blm10 might not be conserved in fruit fly and fission yeast; [65,66]), facilitates nuclear import of the mature CP [48] (Figure 1D). Based on our findings, Blm10 represents the first CP-dedicated nuclear import receptor for the mature CP [48,49]. Blm10 is a 240-kDa protein that belongs to the HEAT-repeat like family and has a similar overall structure as karyopherin β, with a toroidal arrangement of HEAT repeats [67,68]. Like β karyopherins, Blm10 binds FG-rich nucleoporins. Blm10-bound CP is dissociated by Ran-GTP, as expected for an importin-cargo complex, once it encounters Ran-GTP in the nucleus [48]. The conserved C-terminal region of Blm10 is required for nuclear targeting [65,69]. We identified a major Ran-GTP binding site within the *C*-terminal region of Blm10, which currently awaits validation by X-ray structure analysis. As nuclear import of the RP does not depend on Blm10, it either follows the canonical pathway or additional nuclear transport receptors exist that mediate the interaction of the RP with the nuclear pore.

With the identification of Blm10 as a CP-dedicated import receptor, it may even be conceivable that Blm10 facilitates NLS-independent import of CP species during cell proliferation, although the Blm10-bound CP species constitute a considerably smaller fraction of proteasomes in proliferating cells, compared to quiescent cells [51].

Furthermore, Blm10 is preferentially associated with CP with open α ring conformations, which is equivalent to an import-competent conformation. Blm10 is also categorized as a chaperone-like protein, as it controls the late steps of CP maturation and binds to those CP in which the N-termini of α subunits are disordered, leading to open α rings. In a simplified view, Blm10 confers a cap on open α rings and assures latent enzyme activity comparable with the free CP in which the α rings are closed [51]. Open α ring conformations certainly predominate in proteasomal mutants affecting CP maturation [51]. In these mutants, Blm10-association is found with CP precursor complexes, the half-CP, as well as pre-holo-CP and mature CP [49,53]. The binding of Blm10 to these CP conformations may allow an alternative nuclear import pathway that is independent of the canonical import pathway. An important point to note is that Blm10 is not an essential protein. Thus, Blm10-mediated nuclear import of CP complexes is not likely to predominate the canonical import pathway. Our finding that nuclear import of CP is significantly delayed in *blm10∆* cells, once they exit quiescence, argues for the canonical import pathway during cell proliferation. In these mutants, nuclear import of the CP is delayed by the duration required to assemble CP precursor complexes from newly synthesized subunits to provide import-competent CP species [48].

In the case of *BLM10* deletion in a mutant background with deficient CP maturation, the RP takes over the chaperoning function of Blm10 in proteasome assembly [51,70]. Consequently, the RP could mediate nuclear import of the CP in precursor and mature configurations, thus providing an example that redundant pathways for the assembly and nuclear import of proteasomes are in place to complement each other, if one component in this sophisticated system is out of order.

## 9. Do Alternative Import Pathways of Proteasomes Exist in Quiescent Vertebrate Cells?

The plasticity of the import pathway for mature CP becomes evident by studies using reconstituted nuclei based on *Xenopus* egg extracts, the same cell-free reconstitution assay that was used to test the capacity of Blm10 in the nuclear import of mature CP from yeast [48]. Quiescent yeast cells and *Xenopus* eggs are similar in that with the resumption of growth, either signaled by the addition of glucose or by fertilization, the mature CP is immediately imported into the nucleus to promote cell proliferation. In the reconstitution system based on *Xenopus* egg extracts, nuclear import of mature CP depends on the presence of Rpn1 and Rpn2, which are both non-ATPase subunits of the RP base, as well as on Hsp90 and karyopherin β. These proteins form with the mature CP the so-called “20S+” import complex. Although the presence of karyopherin β provides a possible link to the canonical import pathway, nuclear import of the “20S+” species was reported to be independent of Ran-GTPase, suggesting that non-canonical concepts of nuclear import exist in vertebrate systems [71].

## 10. Is There a Common Principle behind the Different Import Pathways of Proteasomes?

So far, all models of import pathways have in common that the CP is not transported as a free particle. Instead, it is either capped by the RP, by Blm10 or is not yet matured, as in the case of inactive precursor complexes. The composition of the nuclear pore selectivity barrier with its intrinsically disordered FG-rich nucleoporins may be incompatible with the transport of free CP, because intrinsically disordered proteins, such as FG-rich nucleoporins, might be preferentially degraded by free CP [21].

## 11. What Is Known about Nuclear Export of Yeast Proteasomes?

In eukaryotic cells, the transition from proliferation to quiescence and *vice versa* is accompanied by profound changes in metabolic pathways [72]. Processes, like trafficking of proteasomes between the nucleus and cytoplasm, as well as dynamic reorganization of hundreds of metabolic enzymes into large proteinaceous, membraneless bodies are observed before cells arrest in quiescence.

With the decline of ATP in quiescence, proteasome holo-enzymes tend to dissociate into RP and CP, which is observed in quiescent yeast [48,73] and neurons with quiescent synapses [74]. In yeast, proteasome holo-enzymes migrate from the nuclear matrix to the nuclear envelope. The significance of these movements may reflect changes in proteolytic demands in these cells. Once the proteasomes are exported through the nuclear pore, they seem to condense into membraneless droplets, which pinch off the nuclear pores and migrate as stabile entities through the cytoplasm. This phenomenon of proteasome droplets in yeast cells was first studied in detail by Isabelle Sagot and her co-workers (2008), who coined the term, proteasome storage granuli (PSG). At the same time, reversible proteasome droplets in the nuclear periphery, named the juxtanuclear quality compartment (JUNQ), were addressed in cell cycle arrested yeast and mammalian cells, suggesting a conserved mechanism underlying proteasome droplet organization [75]. Motile proteasome droplets are also found in the dendrites of primary neuronal cells [76]. In order to understand the organization of proteasome droplets, it is important to decipher the nuclear export of proteasomes, as nuclear export precedes proteasome droplet formation.

However, nothing is yet known about their nuclear export. The most important questions are why proteasomes exit the nucleus during quiescence and how the movement of proteasomes between the nucleus and cytoplasm is regulated, if they are transported as holo-enzymes.

The activity of nuclear proteasomes is regulated by post-translational modifications, such as phosphorylation [77], which may also influence proteasome dynamics. *N*-myristoylation of Rpt2, an RP base ATPase, was reported to regulate proteasome localization. Proteasomes without *N*-myristoylation are not retained in the nucleus and reside in cytosolic droplets of proliferating cells [78]. The RP of proliferating yeast cells in which the bipartite NLS of Rpn2 was deleted also remains in cytosolic droplets [34]. However, cytosolic proteasome droplets formed during cell division and ongoing synthesis of new proteasomal precursor complexes may not be equivalent to the cytosolic proteasome droplets formed in quiescence, when the synthesis of proteasomal precursor complexes is stalled and old proteasomes are sequestered into droplets.

## 12. Conclusions

In this review, I have depicted two models for nuclear import of proteasomes. In one model proteasomes are imported into the nucleus as precursor complexes and modules suggesting that nuclear proteasomes are matured in the nucleus. In this model, nuclear import of proteasomes depends on the canonical nuclear import pathway consistent with the finding that classical nuclear localization sequences are present in several proteasomal subunits. In an alternative model proteasomes are transported into the nucleus as holo-enzymes independent of the canonical import receptor pathway. The mechanism of nuclear export of proteasomes is unknown. Future studies are required to provide insight into which exportins/karyopherins mediate the passage of proteasomes through the nuclear pore during the transition from proliferation to quiescence.

As we improve our understanding of proteasome dynamics and the impact of proteasomal protein degradation in different cellular compartments, our knowledge will significantly influence drug screens for proteasome activators and importers / exporters, which promise to relieve the burdens of unwanted protein aggregations triggered by stress in quiescent cells, especially in non-dividing neuronal cells.

The long-term goal is to gain insight into the enzymology of quiescent cells, how quiescent cells are kept “on call” to respond to extracellular signals and why metabolic enzymes with latent activities, not only the proteasome, are transiently sequestered into reversible protein droplets and reactivated upon request. This type of research has the potential to impact the biomedical study of neurodegenerative diseases and should facilitate the development of next-generation proteasome inhibitors and activators.
